# Impact of COVID‐19 on social prescribing across an Integrated Care System: A Researcher in Residence study

**DOI:** 10.1111/hsc.13802

**Published:** 2022-03-30

**Authors:** Debra Westlake, Julian Elston, Alex Gude, Felix Gradinger, Kerryn Husk, Sheena Asthana

**Affiliations:** ^1^ 6633 Community and Primary Care Research Group Faculty of Health, Medicine, Dentistry and Human Sciences University of Plymouth Plymouth UK; ^2^ Centre for Evidence‐Based Medicine Nuffield Department of Primary Care Health Sciences University of Oxford Radcliffe Observatory Quarter Oxford UK; ^3^ Applied Research Collaboration South West Peninsula (PenARC) Faculty of Health, Medicine Dentistry and Human Sciences University of Plymouth National Institute for Health Research Plymouth UK; ^4^ Plymouth Institute of Health and Care Research Plymouth UK; ^5^ Present address: Centre for Evidence‐Based Medicine Nuffield Department of Primary Care Health Sciences University of Oxford Radcliffe Observatory Quarter Oxford UK

**Keywords:** COVID‐19, Integrated Care Systems, link worker, primary care, researcher‐in‐residence, social prescribing

## Abstract

Emerging evidence suggests that connecting people to non‐medical activities in the community (social prescribing) may relieve pressure on services by promoting autonomy and resilience, thereby improving well‐being and self‐management of health. This way of working has a long history in the voluntary and community sector but has only recently been widely funded by the National Health Service (NHS) in England and implemented in Primary Care Networks (PCNs). The COVID‐19 global pandemic coincided with this new service. There is wide variation in how social prescribing is implemented and scant evidence comparing different delivery models. As embedded researchers within an Integrated Care System in the Southwest of England, we examined the impact of COVID on the implementation of social prescribing in different employing organisations during the period March 2020 to April 2021. Data were collected from observations and field notes recorded during virtual interactions with over 80 social prescribing practitioners and an online survey of 52 social prescribing practitioners and middle managers. We conceptualise social prescribing as a pathway comprising access, engagement and activities, facilitated by workforce and community assets and strategic partnerships. We found that these elements were all impacted by the pandemic, but to different degrees according to the way the service was contracted, whether referrals (access) and approach (engagement) were universal (‘open’) or targeted (‘boundaried’) and the extent to which practitioners’ roles were protected or shifted towards immediate COVID‐specific work. Social prescribers contracted in PCNs were more likely to operate an ‘open’ model, although boundaries were developing over time. We suggest the presence of an explicit, agreed delivery model (whether ‘open’ or ‘boundaried’) might create a more coherent approach less likely to result in practitioner role drift, whilst allowing flexibility to adjust to the pandemic and enhancing practitioner satisfaction and well‐being. The potential consequences of different models are examined.

**What is known about this topic**

- Social prescribing connects people to activities in their community to improve their well‐being. There is no one agreed definition or model.
- Social prescribing has been implemented in NHS primary care; there is limited evidence on implementation in this setting.
- There is little evidence of how social prescribing services have responded to COVID‐19.

**What this paper adds**

- COVID highlighted variability and caused shifts in social prescribing implementation, particularly in primary care.
- COVID changed referrals, social prescribers’ work with clients and availability of activities in the community.
- Primary care social prescribers shifted to COVID‐specific work and supported people with complex needs. Practitioner well‐being was better protected by a ‘boundaried’ model.

## 1 INTRODUCTION

The prescribing of non‐medical support, activities and experiences is spreading around the world as a way of helping some people to manage their illness, improve their health and well‐being and address the wider determinants of health and inequalities (Drinkwater et al., 2019; Munford et al., 2020). These approaches, often labelled social prescribing, are designed to support the non‐clinical needs of people who may need help with their mental health, who are lonely or isolated and who have long‐term conditions or complex social needs (Polley et al., 2017; Tierney et al., 2020). Social prescribing has a long history in the voluntary and community sector (Dayson, 2017; Department Health and Social Care, PHE, & NHSE, 2016) and is a key component of NHS England policy (NHS England, 2019c). There is a financial commitment for every General Practice to have access to a social prescribing ‘link‐worker’ by 2023 (NHSE, 2019b). Denmark, Sweden and Canada are among other countries currently implementing social prescribing (Bhatti et al., 2021; Vidovic et al., 2021).

Social prescribing has been conceptualised as a pathway (Husk et al., 2020) that includes a practitioner who supports people to identify and engage with community‐based activities or experiences (Bertotti et al., 2018; Tierney et al., 2020). In England, this practitioner may be variously titled a Social Prescriber, Social Prescribing Link Worker, Well‐being Coordinator and funded or employed by Primary Care Networks (PCNs), Voluntary, Community and Social Enterprise (VCSE) organisations, Local Authorities or National Health Service (NHS) Trusts (Westlake et al., 2021).

‘Access’, ‘engagement’ and ‘activities’ are key elements of the social prescribing pathway (Westlake et al., 2021). We define *access* as the recipients of social prescribing and the ways they are connected into the service. Social prescribing has been made available to a wide range of client groups, from people experiencing social isolation, loneliness or bereavement, those requiring support to live more active, healthier lives to those who are experiencing financial or relationship stress. *Engagement* concerns what happens in the one‐to‐one interaction with the connecting practitioner. This can vary in frequency, length and content, with a continuum from transactional sign‐posting to services and activities to a holistic, transformative conversation where the practitioner interaction becomes part of the intervention (Kimberlee, 2015). The latter approach is more person‐centred and focussed on defining shared goals and a plan for the client. Social prescribing involves connection to a wide range of *activities* or services, such as interventions focusing on lifestyle or mental health, nature‐ or arts‐based experiences or debt and housing advice.

These three elements are interconnected. For example, the type and content of an interaction with a social prescriber is related to the types of people and needs referred into the pathway, as well as the activities and experiences that are available or known to the social prescriber. The workforce, or practitioner, is an essential asset, enabling this pathway through their skills and experience (Westlake et al., 2021).

The VCSE sector has a key role in building community assets for the pathway (South et al., 2019) situated within a wider context of social, cultural and environmental assets and partnerships (Dayson, 2017). Partnerships between organisations may be directed towards strategic objectives such as addressing health inequalities or comprise organisations and groups working together operationally. Without this wider context of enablers, the capacity, capability and impact of the social prescribing pathway is impeded (Pescheny et al., 2018).

The COVID‐19 pandemic, which impacted England in early 2020, added a new context to existing social prescribing structures. Social distancing and lockdown measures limited citizens’ ability to meet face‐to‐face. The effect of this strategy on the population's overall health and well‐being (aside from infection rates) has yet to be fully investigated (Younan et al., 2020), but local reports showed increased feelings of social isolation and mental ill health for many people (Manion, 2020–2021). The strategy has also affected statutory services’ capacity and ability to deliver. Importantly for social prescribing, many face‐to‐face community activities and groups were suspended (Ogden, 2020; Tierney, 2020).

The coincidence of the pandemic with the early stages of a national rollout of social prescribing in PCNs led to a unique set of circumstances with the potential to impact on implementation of social prescribing. The aim of this paper is to explore how the delivery of social prescribing in the Integrated Care System (ICS) was changed by, or adapted to, the situation under COVID‐19. We look at challenges and opportunities for implementation, how (and if) these were being overcome and future implications for social prescribing.

## 2 RESEARCH DESIGN

### 2.1 Setting

The ICS comprised 131 GP practices in 31 PCNs. PCNs, introduced in 2019, are collaborative networks of general practices serving between 30,000 and 50,000 patients (NHSE, 2019b). By March 2020 PCNs had employed at least one, and in some cases a team, of social prescribers using the NHS funding scheme. However, there was significant variation with respect to employment of social prescribers. Some 40% of PCNs had employed social prescribers themselves, whereas over half had sub‐contracted employment and management to local VCSE organisations, such as regional Councils for Voluntary Service or place‐based partnerships of public/statutory and VCSE organisations (Westlake et al., 2021). Other PCNs had adopted a mixed model in which some practitioners were sub‐contracted from the VCSE sector and others directly employed. In one locality there was no coordinating VCSE organisation; the three PCNs sub‐contracted their social prescribing service to an NHS Trust (acute and community). Remaining districts had previously commissioned VCSE‐employed Well‐being Coordinators (WBCs) who worked within different service boundaries. WBCs continued to receive funding from a variety of sources and overlapped in geography and remit with PCN‐funded staff. In some areas they had been fully or partially sub‐contracted by PCNs under the NHS scheme and retained their title and job role. The management structure and operational characteristics of NHS general practice was very different to the characteristics of VCSE organisations and other NHS organisations such as acute and community trusts. This had significance for the implementation of social prescribing, as we go on to explore.

Evaluation findings represent all these types of employing organisation, although principally respondents were from NHS‐funded schemes operating in PCNs (directly contracted or sub‐contracted). Practice and titles varied (at least 11 titles). For simplicity we call all frontline staff ‘social prescribers’ (SPs) or ‘practitioners’.

The characteristics and culture of these employer organisations, as well as the timeline of employment of the practitioners in relation to COVID, were important contexts. During the first lockdown in March 2020, PCNs were developing (O’Dowd, 2020). Guidance on job descriptions for social prescribing and relationships with primary care colleagues were evolving. In contrast, Well‐being Coordinators, funded through other routes and employed by VCSE, had been in post for some years and had established working practices and connections to their local communities when the pandemic hit.

### 2.2 The Researcher in Residence model

During this study, university‐employed Researchers in Residence (RiRs), based in the ICS, worked with frontline staff, managers and strategic leaders to co‐produce evidence, support service evaluation, improvement and strategic development of social prescribing (Gradinger, 2019; Hazeldine, 2021). Prior to March 2020 they had mapped projects across the ICS footprint and developed trusting relationships with key leaders, VCSE organisations and some primary care‐based social prescribers (Westlake et al., 2021).

COVID constrained the embedded role, but the characteristics of the method enabled data to be generated whilst complying with public health guidelines. The primary RiR worked from home and conducted meetings and interviews using videoconferencing and email. Despite the limitations, the RiR maintained an active participant role, including supporting frontline staff through significant challenges.

### 2.3 Data collection

Qualitative data were collected from 82 participants representing all localities in the ICS: 3 strategic leads, 12 managers, 5 VCSE providers, 5 community builders and 57 frontline social prescribers in VCSE, NHS Trusts and primary care. Sources include formal meeting minutes (*n* = 15), researcher notes from meetings (*n* = 46), VCSE/PCN reports and evaluations (*n* = 11), semi‐structured interviews with VSCE stakeholders (*n* = 9), naturally occurring conversations (*n* = 28) and emails (*n* = 67) with and from front‐line staff and managers. Field notes about meetings and observations captured pertinent verbatim quotes. Analysis of these diverse qualitative data enabled us to check the validity of findings by comparing findings across sources (triangulation).

Quantitative data were also included in an exploratory sequential design method (Cresswell & Plano Clark, 2011) where initial qualitative findings generated questions that could be best answered by quantitative enquiry to establish the impact of COVID on working conditions for social prescribers employed by different organisations. During April–May 2020, the RiRs and ICS delivery team co‐designed and distributed an online service‐evaluation survey to 52 social prescribers (n = 6 managers and 46 frontline staff), representing all PCNs and localities, to investigate their experiences of delivering social prescribing under lockdown restrictions. The survey gathered both quantitative and qualitative data about the availability of support to work remotely provided by different employers, as well as respondents’ perceptions about impact on service users and those delivering services.

### 2.4 Ethics and consent

The University Faculty Ethics Committee Chair confirmed that this was a service evaluation, co‐produced with the ICS, and that ethical approval was not required. Nevertheless, ethical principles were applied to this study. Oral consent was obtained to collect data prior to all online meetings with permission gained for online recording of interviews (Gradinger, 2019). Written consent was gained for survey data collection. Data were held on secure, encrypted computers. Participant and place names were pseudonymised prior to data analysis on a secure, university server, with access restricted to the research team and in compliance with GDPR. Participants were given the opportunity to review the data held and evaluation reports to ensure they were happy with the content and anonymisation process.

### 2.5 Analysis

Qualitative data were managed and analysed using the qualitative data analysis software QSR NVivo 12, and the Framework approach for thematic analysis and cross‐case comparison (Gale et al., 2013; Ritchie & Lewis, 2003). Data were deductively coded by DW and AG according to our research questions and inductively open‐coded according to emerging findings. A sample of data were double‐coded by other team members (JE and FG), followed by subsequent analysis into categories that pertained to social prescribing assets and the pathway (access, engagement and activities).

Quantitative survey data were analysed using Microsoft Excel^®^ to calculate numbers and percentages, and to explore challenges and resourcing issues by role, funding source, employing organisation and locality. Data were integrated sequentially with qualitative data collected from both the survey and other qualitative data sources (Guest & Fleming, 2015).

Continuous sense‐checking of preliminary findings and understandings with practitioners and commissioners informed the iterative process of analysis. Two final workshops were held with the research team and ICS Social Prescribing Executive to frame implications and recommendations for practice using MIRO^®^ and EasyRetro^®^. These online collaboration tools were helpful to encourage co‐production by all stakeholders at a time when face‐to‐face meetings were not possible. Draft summary reports were co‐produced with stakeholders.

## 3 FINDINGS

**FIGURE 1:**
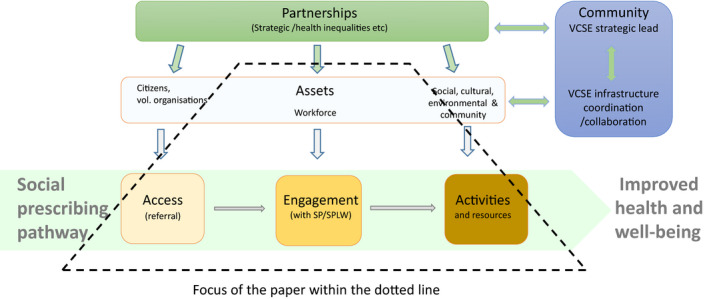
Social Prescribing Framework describing the social prescribing pathway and assets and enablers (Westlake et al., 2021)

COVID had an impact on all elements of our framework for social prescribing (see Figure 1). Here we focus on practitioners as assets and the three elements of the pathway (access, engagement and activities). We explore these in turn, also considering variation in impacts according to the social prescriber's employing organisation, which emerged as a significant theme early in analysis. We also consider the extent to which a delivery model was explicit.

### 3.1 Practitioner assets (the workforce who provide social prescribing)

The pandemic hit just as PCNs were being established and NHS funded and directly contracted social prescribers were being employed. Many social prescribers did not know the primary care teams or their local areas well. Social prescribers funded and contracted by VCSE organisations had been working for longer in these roles and were already familiar with people in their organisations and communities. This gave them a head start in responding to local needs of people who were isolating. They could be flexible in the way they worked in their communities, as their delivery model was well established.

The tools needed for working from home (or virtually) were provided quickly for PCN‐funded prescribers sub‐contracted to other organisations, but more slowly for those directly contracted by PCNs. Most VCSE sub‐contracted social prescribers had been provided with mobiles (85%) and laptops (79%) at the time of the survey, while all working for an NHS Trust had been issued this equipment prior to lockdown. By contrast, just over half of PCN directly contracted practitioners had been provided with laptops and mobile phones by April 2020, and only 33% had online conferencing software (e.g. Microsoft Teams^®^ licences): 44% had remote access to PCN patient‐record systems that allowed them to pick up referrals and contact details for patients and record activity. PCN directly contracted social prescribers reported reluctantly using their own phones to contact clients from home (with associated safeguarding concerns) or, in the absence of designated work‐space, working in out‐buildings or hot desking in surgeries to access phones and record systems:

I spoke to GPs and said I should be able to have access to Zoom and other systems they have but…it hasn’t been an option unless I use my own mobile (SP5 27/08/20).

When asked what was most important to help them do their job remotely during the first lockdown, most frequently mentioned was IT, followed by supervision and guidance for non‐face‐to‐face working. About 79% of VCSE and all NHS Trust sub‐contracted employees could take part in regular online meetings with their team and managers. However, this was only the case for 22% of PCN directly contracted practitioners, many of whom felt they were not yet incorporated in the team: *A few [primary care staff] said they didn't know they employed me*.’ (SP5, 27/08/20). Because of this, some reported experiencing isolation and frustration at not being able to do their job, a particular problem for newly employed social prescribers working alone in GP practices or from home.

### 3.2 Access (who receives social prescribing and how they are referred)

Referrals to all PCN‐funded social prescribing services declined during early phases of the pandemic as GPs were not providing routine appointments, therefore not seeing patients they may have referred. However, after the end of the first lockdown period, referrals and caseloads began to build for most practitioners. At the same time, population needs under COVID brought in a new cohort of people who may not previously have had contact with social prescribing.

PCN directly contracted social prescribing was different to social prescribing in other organisations. Practitioners adopted a responsive approach to who should access the service and how they should be engaged, determined by the preferences of primary care colleagues. This included working with frequent attenders as well as performing COVID‐specific work, such as helping with the vaccination effort. However, this broad service (‘open’ model) was often not explicitly stated or agreed between social prescribers and primary care. A consequence of the ‘open’ model was an increase in referrals with complexity of needs including those with suicidal ideation, trauma or experiencing domestic violence, who had limited access to statutory services under COVID restrictions. Where this model was not explicitly agreed, practitioners often felt overwhelmed by complex clients and ill‐equipped in training and supervision to support them. Social prescribers reported impacts on their health and well‐being; one SP was experiencing feelings of ‘*guilt, segregation, helplessness, anxiety*’ (SP1 24/11/2020).

During the first lockdown, some PCN directly contracted social prescribers were tasked with coordinating COVID‐specific work such as welfare checks for those designated as vulnerable to infection and advised to isolate (the ‘shielding list’, previously known as the Vulnerable Patient List (NHS digital, 2020)). Some found they were duplicating this work with local authority and VCSE colleagues. These contacts increased referral numbers to social prescribing, although most clients were not subsequently enrolled into the social prescriber caseload.

In contrast, VCSE and NHS Trust contracted staff were more likely to operate within an explicit, agreed delivery model in which referral criteria were targeted to certain populations – either those within defined communities or certain cohorts felt to benefit most from social prescribing: for example, people aged over‐55 and isolated or those who were ‘*ready to work’* on coaching goals and make lifestyle changes. This more ‘boundaried’ model of social prescribing sometimes excluded those experiencing severe mental health crises. Practitioners were better protected from ‘access shift’ as they operated within agreed and explicit boundaries and had established pathways (e.g. good relationships to referrers, clients and community assets). In some cases, this led to a decline in referrals and caseload during COVID. Some VCSE organisations experienced pressure from their primary care funders to accept referrals of individuals with high levels of social and mental health complexity that they felt were outside their remit; in one case this led to withdrawal from the contract.

### 3.3 Engagement (the interaction with the social prescriber)

As lockdown was introduced, engagement shifted to non‐face‐to‐face modes of contact. SPs across organisations reported that many clients lacked digital access or preferred to engage via telephone so few were using video conferencing to speak to clients.

PCN directly contracted practitioners typically delivered the ‘open’ model. During the pandemic, the nature and content of engagement shifted towards more sign‐posting (transactional) interactions for some clients. This was because of a lack of ongoing community activities to refer into and the growing demands of COVID‐specific work, such as welfare checks to signpost shielding people to local community services that would deliver food, prescriptions and other support: ‘*We've had to call all the shielding list which started off at 564 and with additions went to 1200*’ (SP2, 03/06/2020). Some PCN directly‐contracted SPs saw this as enhancing their status as primary care team members ‘*all doing it together*’ (SP03, 11/02/21) or considered it a ‘*privilege’* to collaborate with the vaccination effort (SP11, 11/02/21). They also noted that working at vaccination centres highlighted unmet needs in the population such as loneliness, particularly with elderly cohorts.

Others felt COVID work was not their role. However, because there was not an explicitly agreed delivery model in primary care, they were not able to challenge this work: ‘*I think it reflects generally how unseen and undervalued social prescribing is’ (SP76 21/4/21)*.

Some PCN directly contracted social prescribers were working over 50% of their week on administrative tasks in vaccination clinics. This meant reducing their usual client‐caseload, introducing waiting lists for referrals or shortening frequency of contacts, thereby limiting their capacity to engage in a holistic or transformational way: ‘*it makes it more difficult to support/encourage progress as contact is less frequent.’ (SP80, 22/4/21)*.

Conversely, for people with complex needs who had nowhere else to go for support, PCN directly contracted practitioners found the suspension of face‐to‐face activities and services led to their engaging in ‘holding’ mental health support calls. These calls could be long, difficult and emotionally draining for social prescribers who found themselves drawing on life skills (and, in some cases, previous professional experience in mental health) to engage with distressed individuals. This represented a significant ‘role drift’ towards a clinical type of intervention they felt ill‐prepared for: ‘*the role has become something that I didn`t sign up for and I`m feeling burned out*’ (SP49, 07/09/2020). During the pandemic, some started to think about drawing boundaries around their work.

VCSE‐contracted practitioners with a remit to work in a holistic way continued to do so as they adapted to pandemic circumstances. Shifts in the content of engagement were less common where there was a clear, agreed model to work to and role boundaries. The NHS trust service adjusted boundaries by offering short, one‐off coaching calls for existing clients who were unable to do the usual activities, such as going to the gym, and for vulnerable cohorts referred due to the pandemic. Nevertheless, the service maintained clear objectives of goal‐oriented work using a motivational approach:

Up to now [we] only enrolled people who could meet goals during the current climate—as of July [we] will take people on for full coaching enrolment if things [activities] are open (SP79, 17/06/20).

### 3.4 Activities (people are connected to by the social prescriber)

Lack of face‐to‐face activities affected all social prescribing, regardless of the employing organisation. Many community activities and groups were subject to cycles of suspension and reopening in compliance with social distancing measures. Some VCSE organisations, who traditionally provide these community assets, re‐directed efforts to welfare support, while some closed down permanently as a result of funding shortfalls prompted by the pandemic. This had significant impact on activities to which social prescribing could connect people in the third step of the social prescribing pathway.

However, there were some differences. Not all PCN directly contracted practitioners were well connected with their local community organisations at the time of the first lockdown, so were less able to respond quickly by connecting people into local resources. Social prescribers found themselves becoming the activity or whole solution for people with severe mental health problems: ‘*there are not the resources in mental health services to meet demand. If the resources were there our jobs would be much easier*’ or working on social issues such as housing, when statutory services could not meet needs: ‘*Ideally it would be good to be able to pass on to a specialist agency / charity but at the moment organisations are so busy it's not always easy to find one to take a referral* (SP66, 17.03.21). Practitioner peer support groups, bringing together SPs from different employing organisations, enabled the sharing of online resources. As adaptations to pandemic‐working progressed, some were able to work alongside VCSE response networks.

Practitioners employed by VCSE organisations responded quickly to the pandemic. They did what they knew best and connected into their local community response:

We have linked very closely with [community organisation] to fully support the COVID‐19 helpline and became the more complex support for the line which plays to the team’s strength in mental health, domestic abuse and suicide and bereavement support. (SP20, 15/05/21).

Some collaborated in community‐building activities: memory cafes, food delivery services, craft groups. The NHS trust service adapted goals to non‐face‐to‐face activities (e.g. walking alone or with one other person instead of going to the gym).

## 4 DISCUSSION

COVID affected all three elements of the social prescribing pathway (access, engagement and activities) and practitioners as key assets. Impact on social prescribing services was significantly influenced by the contracting organisation and whether a clear, agreed delivery model was in place. Services demonstrated a continuum between more ‘open’ and ‘boundaried’ approaches and more explicit and implicit delivery models (see Appendix for case studies of the ‘open’ and ‘boundaried’ models). In the context of COVID, this impacted on who was referred, how they were engaged and practitioner health and well‐being.

These findings resonate with other studies that have conceptualised social prescribing as varying in key dimensions along a continuum or spectrum of delivery (Calderón‐Larrañaga et al., 2021). Kimberlee suggests a classification of ‘light’ (mostly signposting) to ‘holistic’ interactions with clients (Kimberlee, 2015).

Existing studies have largely focussed on our *engagement* element of the pathway, and have not extended analysis to the interaction between other pathway ingredients, nor to consequences for the practitioners. Calderón‐Larrañaga has defined ‘good’ social prescribing as an open–ended, ongoing interaction between client and prescriber, responsive to changes in client circumstances over time (‘relational’ archetype). This is opposed to more ‘transactional’ relationships with pre‐established limits on number of sessions and type of interaction.

These social prescribing archetypes do not map exactly to our ‘open’ and ‘boundaried’ models, nor to light versus holistic, but there are overlaps. The ‘relational’ archetype has more in common with our ‘open’ model, but we argue that the designation of ‘good’ social prescribing does not take into account the consequences for sustainability if practitioner well‐being is not maintained. There are implications of ‘open’ referral (*access*) for the support and training needed to engage with, for example, someone with severe mental health concerns and the length and content of *engagement* required to make a difference. Most importantly, the position of a service along these continua must be shared, explicit and *agreed* with the contracting organisation and the primary care teams. ‘Open’ model practices were not explicitly agreed upon as the delivery model of social prescribing, so tended to have far looser referral criteria and lack of understanding of the implications for practitioner engagement.

Within the context of the pandemic, there were further disadvantages to the more ‘open’ model, including *role drift* that left practitioners feeling confused and over‐burdened. They were unsure of their remit and concerned that they were not trained to work with all client groups, a finding also identified in a study in Scotland (Fixsen et al., 2021). Often because of COVID‐specific work and caseload pressures, practitioners were less able to maintain a personalised or ‘holistic’ model of social prescribing and more likely to shift towards simple signposting.

The employing organisation appeared to have an important influence on the objectives and outcomes of all three elements of the service; whether they were ‘open’ or ‘boundaried’, implicit or explicit or somewhere in between, as well as the level of practitioner training and support on offer to deliver the model. However, the extent to which this is a function of the employing organisation itself or the timing of the PCN roll‐out of social prescribing is debatable (Norman et al., 2021). By contrast, Well‐being Coordinators funded through other routes and employed by VCSE organisations had been in post for some years and had established working practices, including development of specialist roles (eg End‐of‐Life or dementia care). They were well supported and connected to their local communities as the impact of the pandemic hit.

As the pandemic recedes and training, supervision and local connections become embedded in PCN‐employed services, it is plausible that staff will be less likely to experience ‘role drift’ and more likely to return to more holistic, person‐centred ways of working. Social prescribing pathways are not fixed, but develop in response to contexts, such as the COVID pandemic and the availability of, and connection to, community assets.

### 4.1 Implications for delivery

COVID has placed unprecedented demands on services, including primary care, and has accentuated barriers and facilitators to the delivery of social prescribing.

1. *Access*: COVID is likely to have a long legacy in terms of mental health, social isolation and economic disadvantage so an increase in demand is to be expected. Social prescribing services need to agree their target audience (volume and suitability) and approach (adequately trained practitioners for their complex and demanding role) and ensure that they do not exclude or widen health inequalities (Fixsen et al., 2021; Gibson et al., 2021; Wildman et al., 2019).
2. *Engagement*: services must ensure SPs are not pulled into other primary care business as the recovery progresses and can return to more transformative or holistic ways of engaging with people. Video conferencing may have a role to play, but face‐to‐face engagement is likely to be more important for some populations, and necessary to avoid digital exclusion. With the roll out of health coaches and care coordinators in primary care (NHS England, 2021), the need for services to clearly define role boundaries, including referral criteria and how clients should be managed, will become increasingly pertinent.
3. *Activities*: as services move to recovery, ICSs will need to invest in their VSCE sector, financially weakened by the pandemic (National Council for Voluntary Organisations, 2020). COVID reinforced the importance of community assets and access to appropriate statutory services. Without these assets, practitioners are liable to become a mental health ‘holding’ service rather than a transformational service.

### 4.2 Strengths

These are contextually rich data collected during a period of unparalleled changes to services and also at a formative time for development of social prescribing in the NHS. Various data types, seen through the lens of multiple actors at different levels of a system, provide triangulation. There is little other real‐time research we are aware of being undertaken during these events. We have shown that our framework for social prescribing is useful in capturing barriers and facilitators to the implementation of social prescribing, and for a contextual analysis of factors such as employing organisation and the COVID pandemic. The framework also facilitated the identification of an important dimension of social prescribing: service models based on a continuum of ‘open’ to ‘boundaried’. Our study presents important implications for implementation and policy at a critical time as ICS organisations are forming and developing policy throughout England (NHS England, 2018, 2019a).

### 4.3 Limitations

COVID constrained the gathering of primary care clinician and service user perspectives. These views are essential to fully understand the impact of COVID and, in different times, these would have been sought. There were variations in service reporting, social prescribing referral and activity data being collected, in part due to there being no agreed definition of social prescribing, nor the role of practitioners.

Some social prescribers did not have access to primary care electronic systems. As a service evaluation with a co‐produced, flexible protocol facilitated by the RiR model, data collection was opportunistic, drawn from multiple systems with a limited budget and timescale. All data types are subject to biases and the normative thinking or opinions of the interviewee, report writer or researcher.

## 5 CONCLUSIONS

Situating social prescribing in primary care organisations where there is not an agreed delivery model is precarious. The evidence presented in this paper suggests an agreed, more ‘boundaried’ model can support social prescribing staff to understand their role and protect practitioner well‐being. Practitioners are empowered to communicate their service clearly to others such as referrers, avoiding the risk of ‘role drift’ while also maintaining practitioner integrity. However, this model may have consequences for health inequalities, as people who have complex social situations or mental health conditions may be excluded by such protective boundaries. This could be mitigated by specialist training, development of roles within primary care and researching impacts of recent PCN incentivisation through the Investment and Impact Fund (NHS, 2021). Practitioners working with people who have complex problems need specialist supervision and both managerial and peer support. This is currently lacking in the NHS model for social prescribers directly employed by PCNs.

## CONFLICT OF INTEREST

There are no conflicts of interest.

## AUTHORS’ CONTRIBUTION

All authors were involved in the conception and design of the study. DW, AG and JE undertook data collection. DW, AG, JE and FG undertook analysis. All authors were involved in the process of interpreting the data. DW prepared the manuscript. All authors have read, made amendments to, and approved the content of the final version of the manuscript.

## Supporting information

Appendix

## Data Availability

The data that support the findings of this study are available from the corresponding author, DW, upon reasonable request.
