# Supplementary figures and images for: Impact of COVID‐19 on social prescribing across an Integrated Care System: A Researcher in Residence study
Source: Health Soc Care Community. 2022 Mar 30:10.1111/hsc.13802. Online ahead of print. doi: 10.1111/hsc.13802 (PMC9111657; doi:10.1111/hsc.13802)

# Case study 1. Impact of COVID on the 'Boundaried' model

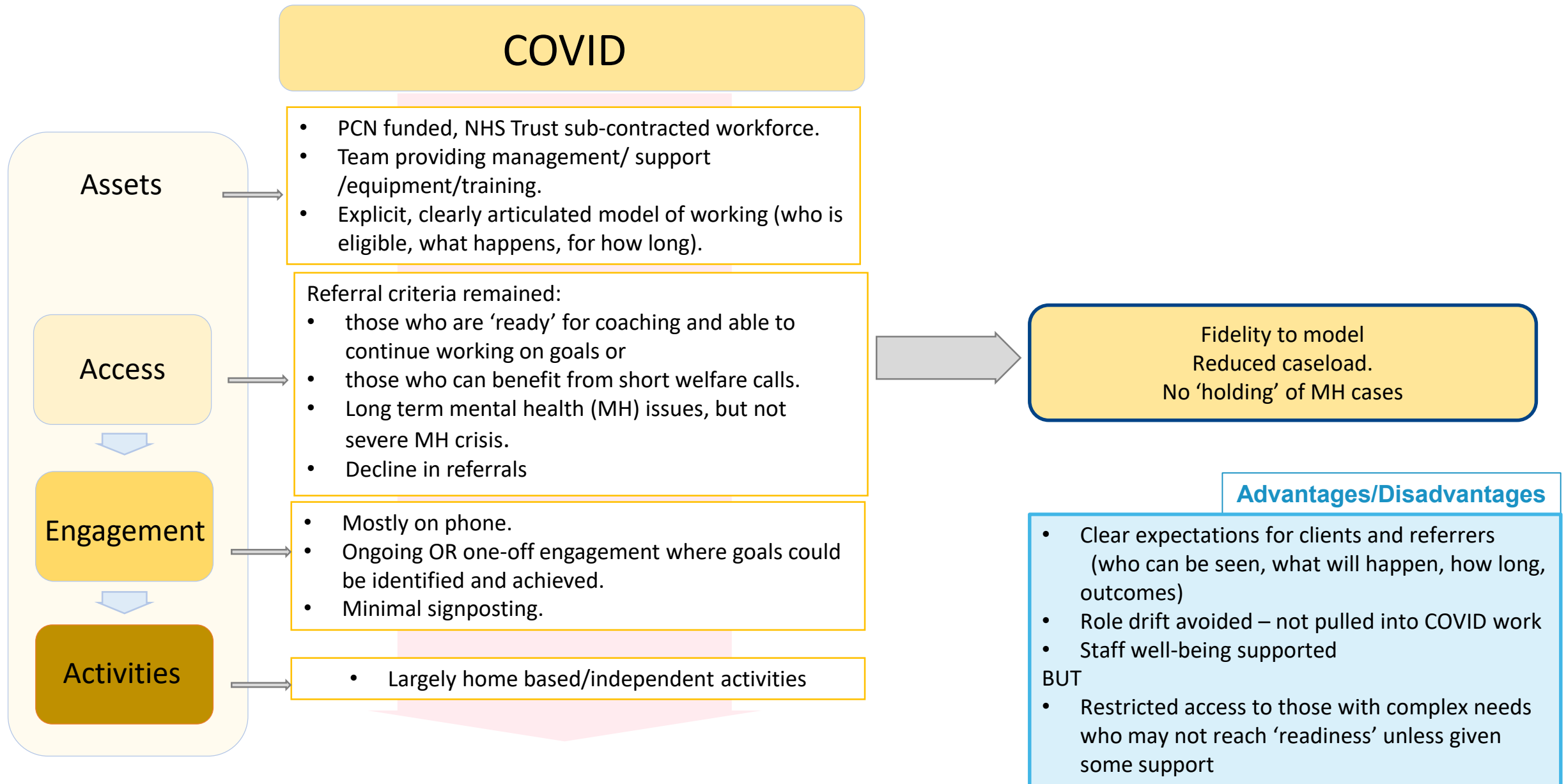

# Case study 2. Impact of COVID on the 'Open' model

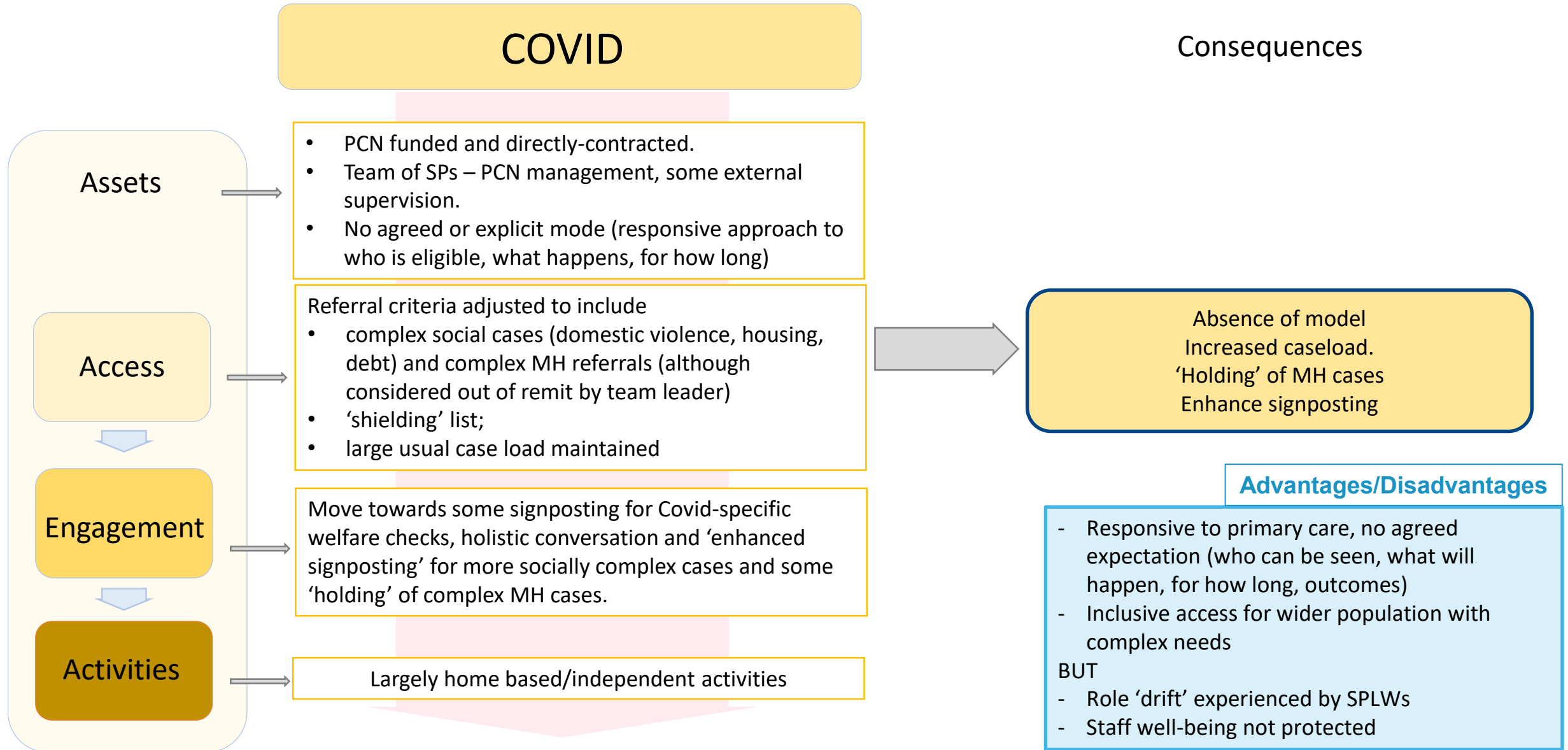

Supplement: Supplementary file 1 — Appendix [file HSC-9999-0-s001.pdf]
